# Sanguinarine Protects Channel Catfish against *Aeromonas hydrophila* Infection by Inhibiting Aerolysin and Biofilm Formation

**DOI:** 10.3390/pathogens11030323

**Published:** 2022-03-07

**Authors:** Lushan Zhang, Liang Ma, Qiuhong Yang, Yongtao Liu, Xiaohui Ai, Jing Dong

**Affiliations:** 1 Yangtze River Fisheries Research Institute, Chinese Academy of Fishery Sciences, Wuhan 430223, China; zhanglushan1993@163.com (L.Z.); maliang199501@163.com (L.M.); yangqh@yfi.ac.cn (Q.Y.); liuyt@yfi.ac.cn (Y.L.); 2 College of Fisheries, Huazhong Agricultural University, Wuhan 430072, China

**Keywords:** sanguinarine, *Aeromonas hydrophila*, antibacterial, virulence factors

## Abstract

*Aeromonas hydrophila* is a pathogenic bacterium that can cause serious infections both in humans and aquatic animals. Antibiotics are the main approach for fighting against the pathogen. However, the emergence of antibiotic resistance has resulted in treatment failure. Therefore, drugs with novel strategies need to be developed. Quorum sensing has been recognized as a promising method for identifying anti-virulence drugs against bacterial infections. The aim of this study was to identify novel drugs targeting quorum sensing of *A. hydrophila* as alternatives of antibiotics in aquaculture. Thus, hemolytic activity, biofilm formation, qPCR and experimental therapeutics assays were conducted. The results showed that sanguinarine inhibited the growth of *A. hydrophila* at concentrations higher than 16 μg/mL, but the production of aerolysin and biofilm formation was significantly inhibited at sub-inhibitory concentrations by disrupting the quorum sensing system. Cell viability results showed that sanguinarine could provide protection for A549 cells from aerolysin-induced cell injury. In addition, the mortality of channel catfish administered with sanguinarine at a dosage of 20 mg/kg decreased to 40%, which showed a significant decrease compared with fish in positive group. Taken together, these findings demonstrated that anti-virulence strategies can be a powerful weapon for fighting against bacterial pathogens and sanguinarine appears to be a promising candidate in the treatment of *A. hydrophila* infections.

## 1. Introduction

*Aeromonas hydrophila* (*A. hydrophila*) is a bacterium, widely distributed in aquatic environments, which can cause a wide range of infectious diseases in aquatic animals. Pathogenic *A. hydrophila* is the main cause of bacterial infections in freshwater fisheries in China. *A. hydrophila*-associated infections have brought huge economic losses in aquaculture, and have seriously restricted the sustainable and healthy development of the industry [1]. Furthermore, *A. hydrophila* has become a zoonotic bacterial pathogen because it can transmit from diseased fish, polluted water and under-cooked seafood to humans by consumption routes [2]. Antibiotics are the main strategy in the fight against bacterial infections in both human and animals [3]. However, the abuse of antibiotics results in the emergence of bacterial resistance [4]. Therefore, drugs with novel targets are urgently needed.

The pathogenesis of *A. hydrophila* is composed of a number of virulence factors, including hemolysin, aerolysin, enterotoxin and adhesin [5,6,7,8]. Aerolysin, produced as an inactive precursor, has been proven to play a critical role in the pathogenesis of *A. hydrophila* [9]. Activated aerolysin exhibited hemolytic, enterotoxic and cytotoxic activities after cleaving 43-aa residues in C terminus by trypsin or furin protease [9]. A number of mammalian cells are sensitive to the toxin [10]. Moreover, strains lacking the aerolysin encoding gene showed a significant reduction in virulence [11]. Thus, aerolysin has been identified as a marker of pathogenic *A. hydrophila* strains. The formation of bacterial biofilms results in antibiotic resistance, and is a source of iterative and continual infections in aquatic animals. Therefore, biofilm formation has been identified as a virulence factor in pathogenic bacteria [12]. Aerolysin and biofilm are regulated by a quorum sensing system. Thus, quorum quenching has been identified as a mechanism for developing drugs against bacterial infections in aquaculture [13].

**Figure 1:**
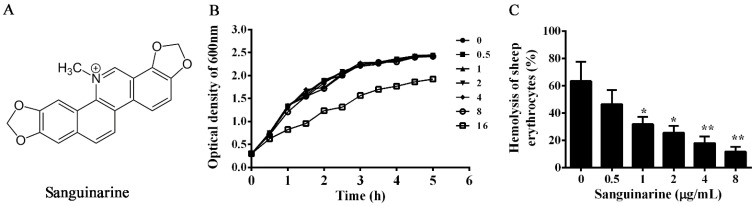
Inhibitory effect of sanguinarine on hemolytic activity of bacterial supernatants. (**A**) Molecular structure of sanguinarine. (**B**) Growth curves of sanguinarine on *A. hydrophila* XS-91-4-1 strain. (**C**) Hemolytic activities of the *A. hydrophila* supernatants co-cultured with different concentrations of sanguinarine. Moreover, 1% Triton X-100 was served as the 100% hemolysis control. All samples were determined in triplicate and data are presented as mean value ± SD. *, *p* < 0.05 and **, *p* < 0.01 when compared with the sanguinarine-free supernatants.

For decades, natural products have represented a source of compounds with biological activities in drug discovery [14]. In China, herbal medicines have a history of thousands of years, and have been widely used in the treatment of infectious diseases in humans, terrestrial animals and aquatic animals [15]. Plant extracts containing a variety of compounds showed antibacterial, anti-inflammatory and antioxidative effects [16]. Sanguinarine (Figure 1A), isolated from *Macleaya cordata* (Willd.) R. Br., has antibacterial, anti-inflammatory, anti-tumor, immunity enhancement and insecticidal effects [17]. Previous studies have demonstrated that sanguinarine can inhibit the pathogenesis of several bacterial pathogens by different mechanisms [18,19,20]. However, the inhibitory effect of sanguinarine on *A. hydrophila* virulence has not been clarified. Therefore, the present study aimed to clarify the inhibitory effect of sanguinarine against *A. hydrophila* virulence. 

## 2. Material and Methods

### 2.1. Bacteria Strain and Drug Preparation

*A. hydrophila* XS-91-4-1 was kindly provided by Prof. Aihua Li of Institute of Hydrobiology, Chinese Academy of Sciences. Sanguinarine (CAS No. 5578-73-4) with a purity of 98% was a commercial product of Chengdu Must Bio-Technology CO., LTD (Chengdu, China), while enrofloxacin (CAS No. 93106-60-6) with a purity of 99% was purchased from the National Institute for Food and Drug Control (Beijing, China). Sanguinarine and enrofloxacin (HPLC ≥ 99%) were dissolved in dimethyl sulfoxide (DMSO) to make a stock solution of 40.96 mg/mL. For in vivo study, sanguinarine was dissolved in 30% propanediol.

### 2.2. Determination of Minimal Inhibition Concentrations (MICs)

The MICs of sanguinarine and enrofloxacin against *A. hydrophila* XS-91-4-1 were evaluated using the broth micro dilution method, as described by Abdelaziz et al. [21]. Sanguinarine and enrofloxacin were diluted in a 96-well plate by a 2-folder method to obtain final concentrations ranging from 64 to 0.5 μg/mL. Then, bacteria were re-suspended in MHB medium and added to each well at a final density of 5 × 10^5^ CFU/mL. The plate was incubated at 28 °C for 16–18 h. The MICs were defined as the lowest concentration at which resulted in no visible growth.

### 2.3. Growth Curves Assay

Bacterial cultures with different concentrations of sanguinarine were co-cultured to determine the effect of sanguinarine on bacterial growth [22]. Briefly, *A. hydrophila* strain XS-91-4-1 was cultured in LB medium at 28 °C to obtain the optical density at 600 nm (OD_600nm_) of 0.3, and the cultures were aliquoted into five flasks in a volume of 10 mL. Then, sanguinarine at final concentrations of 0.5, 1, 2, 4, 8 and 16 μg/mL was added to each flask. DMSO was added to the drug-free group to determine the role of DMSO on bacterial growth. The mixtures were further cultured at 28 °C with constant shaking for 5 h, and bacterial growth was monitored by reading the OD_600nm_ values every 30 min.

### 2.4. Hemolytic Activity Assay

Hemolytic activity was measured as described previously [23]. XS-91-4-1 strain was cultured in LB medium with indicated concentrations of sanguinarine to obtain the OD_600nm_ of 1.5. Then, the supernatants of the bacterial cultures were harvested by centrifugation and activated by trypsin. The hemolytic reaction system was brought up by 875 μL hemolytic buffer (20 mmol/L Tris, 150 mmol/L NaCl, pH 7.2), 100 μL bacterial supernatant and 25 μL defibrillated sheep erythrocytes. The mixtures were then incubated for 15 min at 37 °C. Unlysed cells were removed by centrifugation. The hemolytic activity was determined by measuring the optical density at 543 nm, and 0.1% Triton X-100 served as the 100% hemolysis control. 

### 2.5. Immunoblotting

The bacterial supernatants collected previously were boiled with loading buffer for 10 min, and 20 μL of the samples were loaded into 12% SDS-PAGE. Then, proteins were transferred onto PVDF membranes (Roche, Basel, Switzerland) using a semi-dry transfer cell (Bio-Rad, Munich, Germany) for 90 min. The membrane was blocked for 2 h with 5% skimmed milk at 37 °C. Subsequently, a primary anti-aerolysin polyclonal antibody at 1:2000 dilution was incubated for 1 h at 4 °C, followed by an additional incubation for 1 h with a HRP-conjugated secondary goat anti-rabbit antiserum. The blot was detected using Amersham ECL Western blotting detection reagents (GE Healthcare, Stockholm, Sweden).

### 2.6. Biofilm Formation Assay and Microscopic Analysis

Biofilm formation was measured in a 96-well plate as described previously with slight modifications [24]. In brief, an overnight bacterial culture was diluted to 1:10 in fresh medium and added to each well of a 96-well plate. Then, sanguinarine was added to each well to obtain final concentrations of 0, 0.5, 1, 2, 4 and 8 μg/mL. Following a further incubation at 28 °C for 24 h without shaking, the planktonic cells were removed by washing twice with PBS. Then, the plate was incubated with 0.5% crystal violet for 30 min after being air-dried. After washing, 30% glacial acetic acid was added to each well to release the crystal violet attached in bacterial cells. The inhibitory effect of sanguinarine on biofilm formation was determined by measuring the absorption at OD_570nm_. DMSO served as the positive control and LB medium was used as the negative control. 

For light microscopic visualization of biofilms, the bacterial cells were allowed to grow on glass slides in a 24-well plate plus indicated concentrations of sanguinarine at 28 °C for 24 h, according to a previous study [25]. The glass slides were washed with PBS three times and air-dried. Then, bacterial cells in the glass slides were stained with 0.5% crystal violet, mounted on a microscopic slide and visualized under a light microscope at a magnification of 400×.

### 2.7. qPCR Assay

Total RNA was extracted from bacterial precipitations using the TaKaRa MiniBEST Universal RNA Extraction kit (TaKaRa, Kyoto, Japan). The purity and concentration of total RNA were measured by a NanoDrop 2000 spectrophotometer (Thermo Scientific, Waltham, MA, USA). The expression levels of aerolysin encoding gene *aerA*, quorum sensing genes *ahyI* and *ahyR* were detected, 16 s rRNA was used as an internal control to normalize the expressional levels. The sequences of primers are listed in Table 1. The qPCR was carried out in a 20 μL total volume with TB Green *Premix Ex Taq* II according to the directions supplied by the manufacturer. The reaction cycles were performed as follows: 95 °C for 30 s; 40 cycles at 95 °C for 5 s; 55 °C for 30 s; 72 °C for 1 min; and 72 °C for 4 min. 

### 2.8. Cytotoxicity Assays 

A549 human lung epithelial cells were cultured in DMEM medium containing 10% bovine fetal serum with 5% CO_2_ at 37 °C. Cells were digested and seeded into a 96-well plate at a density of 5 × 10^4^ cells per well. Cells were incubated with the presence of bacterial supernatants prepared with indicated concentrations of sanguinarine for 2 h at 37 °C. Cell viability was determined by live/dead assays, cells after treatment with bacterial supernatants were incubated with live/dead regents for 30 min, and images were captured by a fluorescence microscope (Olympus, Tokyo, Japan).

### 2.9. Experimental Therapeutics

Animal studies were performed under the guidance of the Animal Welfare and Research Ethics Committee at Yangtze River Fisheries Research Institute (Permission No. YFI-2020DJ-011, 6 May 2020). All the experimental protocols were approved and supervised by the animal care committee. Fish were obtained from our breeding center, and 30 channel catfish weighing 100 ± 5 g were separated into 3 experimental groups at 1 m^3^ glass tanks with aeration at 28 °C. The dissolved oxygen was maintained 6 mg/L and the pH of water ranged from 7.2 to 7.4. An overnight bacterial culture was centrifuged to collect the cells, then re-suspended twice in PBS. The bacterial suspension was adjusted to 1.5 × 10^8^ CFU/mL using McFarland standards. Then, fish were infected with *A. hydrophila* by injecting 100 μL of bacterial suspension intraperitoneally per fish. Fish were given 20 mg/kg sanguinarine suspension using a gavage needle post-infection and at 12 h intervals for 3 days. Fish in the positive group were given sterile PBS after being infected with *A. hydrophila*, while fish in the negative group were injected with sterile PBS and administered with PBS at the same intervals as the sanguinarine-treated group. Fish in each group were fed with normal diet, and deaths in each group were observed for 8 days. 

### 2.10. Statistical Analysis

Data were analyzed using independent Student’s t-test by SPSS 20.0 statistical software (SPSS Inc., Chicago, IL, USA). The survival rate was analyzed using the Kaplan–Meier estimates method, and the significance of different groups was analyzed with the log-rank test. A *p* value < 0.05 was considered to be statistically significant. 

## 3. Results

### 3.1. The Effect of Sanguinarine on the Growth of A. hydrophila

The MICs of sanguinarine and enrofloxacin against *A. hydrophila* XS-91-4-1 strain were 32 μg/mL and 4 μg/mL, respectively. Moreover, the influence of sanguinarine on the growth of *A. hydrophila* was determined. As shown in Figure 1B, sanguinarine had no impact on the growth of *A. hydrophila* in 5 hs at concentrations ranging from 0.5 to 8 μg/mL. However, we found that the growth of the bacterium plus 16 μg/mL sanguinarine was much lower than that of 8 μg/mL. These results indicated that there was no influence of sanguinarine on bacterial growth at concentrations lower than 16 μg/mL.

### 3.2. Sanguinarine Decreased the Hemolysis of Bacterial Supernatants by Inhibiting the Expression of Aerolysin

**Figure 2:**
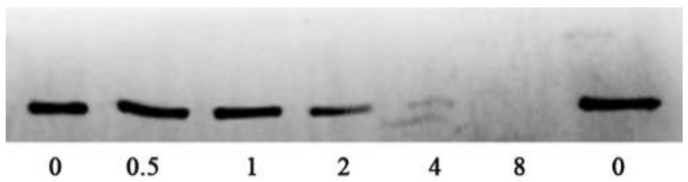
Immuno-blot of aerolysin in bacterial supernatants.

The hemolytic activities of bacterial supernatants were detected by defibrillated sheep erythrocytes. As shown in Figure 1C, the hemolytic activities were decreased in a dose-dependent manner. Statistical significance was observed in hemolytic activities at concentrations higher than 0.5 μg/mL. When treated with 8 μg/mL sanguinarine, the hemolytic activity was reduced to 17.97%. Moreover, Western blot assay was carried out to clarify the impact of sanguinarine on aerolysin production in the supernatants. As desired, the productions of aerolysin in the supernatants were reduced by the addition of sanguinarine (Figure 2). Taken together, these results demonstrated that sanguinarine could reduce the hemolytic activities of bacterial supernatants at sub-inhibitory concentrations by inhibiting the production aerolysin.

### 3.3. Sanguinarine Decreased the Biofilm Formation of A. hydrophila

**Figure 3:**
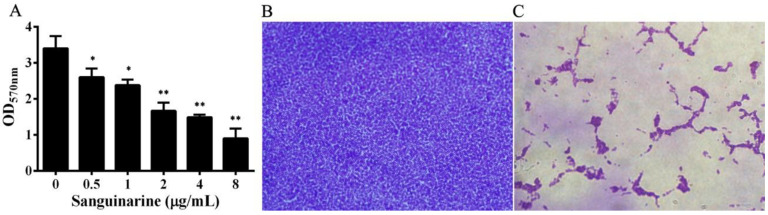
Sanguinarine reduces biofilm formation of *A. hydrophila*. (**A**) Determination of biofilm co-cultured with indicated concentrations of sanguinarine, data in (**A**) represented mean ± SD of three independent assays, *, *p* < 0.05 and **, *p* < 0.01. (**B**,**C**) microscopic validation of biofilm formation co-cultured with sanguinarine, (**B**) drug-free group; (**C**) *A. hydrophila* co-cultured with 8 μg/mL sanguinarine.

The inhibitory effect of sanguinarine on biofilm formation was determined. As shown in Figure 3A, sanguinarine treatment could reduce the biofilm formation of *A. hydrophila* in a dose-dependent manner. The amount of biofilm reduced to 77.39 ± 14.67%, 70.63 ± 11.28%,49.49 ± 9.60%, 43.89 ± 2.69%, 26.14 ± 5.97% when treated with sanguinarine at concentrations of 0.5, 1, 2, 4 and 8 μg/mL, respectively. Moreover, biofilm formation was determined microscopically on glass slides with indicated concentrations of sanguinarine. As expected, a dense biofilm was observed on drug-free glass slides (Figure 3B), while a visible reduction in biofilm occurred on the glass slide treated with 8 μg/mL sanguinarine (Figure 3C).

### 3.4. qPCR Results

**Figure 4:**
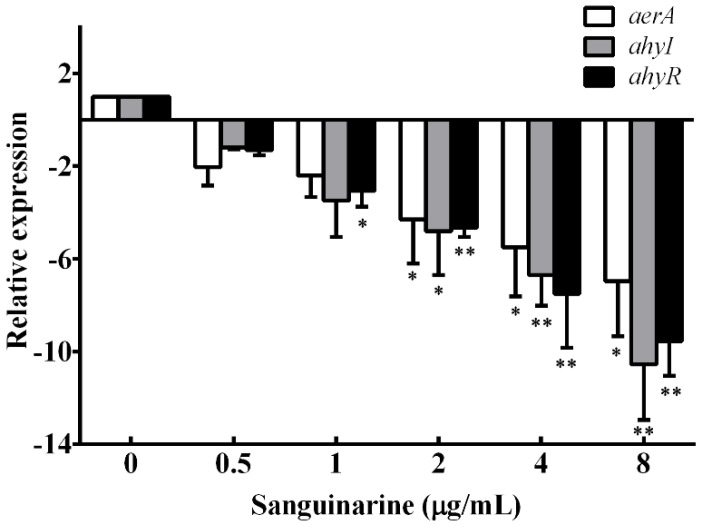
Relative expression of related genes. All samples were determined in triplicate and data are presented as mean value ± SD. *, *p* < 0.05 and **, *p* < 0.01 when compared with the sanguinarine-free group.

The results shown above indicate that sanguinarine might affect the transcription of the quorum sensing system. Therefore, qPCR assay was conducted to analyze the influence of sanguinarine on the aerolysin encoding gene *aerA* and quorum sensing genes *ahyI* and *ahyR*. As shown in Figure 4, the transcription of *aerA*, *ahyI* and *ahyR* genes was inhibited following the increasing concentrations of sanguinarine. The *aerA*, *ahyI* and *ahyR* genes were remarkably down-regulated when the concentration of sanguinarine reached 2 μg/mL and above compared with the drug-free group (Figure 4). The result revealed that sanguinarine could reduce the production of aerolysin and biofilm formation by inhibiting the quorum sensing system.

### 3.5. Sanguinarine Protected A549 Cells from Aerolysin Mediated Cell Injury

**Figure 5:**
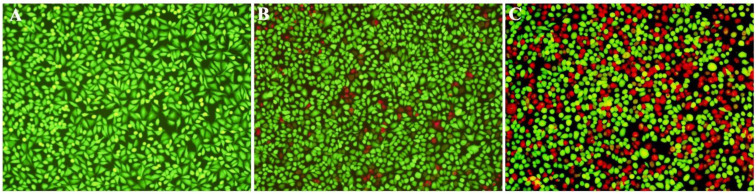
Sanguinarine protects A549 cells from aerolysin-induced cell injury. (**A**) Uninfected cells; (**B**) cell-incubated supernatant plus 4 μg/mL sanguinarine; (**C**) co-cultured with *A. hydrophila* strain XS-91-4-1 without sanguinarine.

Live/dead staining was performed to evaluate the protective effect of sanguinarine on A549 cells against aerolysin-mediated cell injury. As shown in Figure 5A, live cells without bacterial supernatant were stained with green. Cells co-cultured with drug-free supernatant were dead and were stained with red (Figure 5C). Cells incubated with supernatant plus 4 μg/mL sanguinarine showed a decrease in cell death compared with the drug-free group (Figure 5B). The results demonstrated that a decrease in aerolysin production in the supernatants could alleviate the cell injury of A549 cells.

### 3.6. Sanguinarine Provided a Protection to the Channel Catfish against A. hydrophila Infection

**Figure 6:**
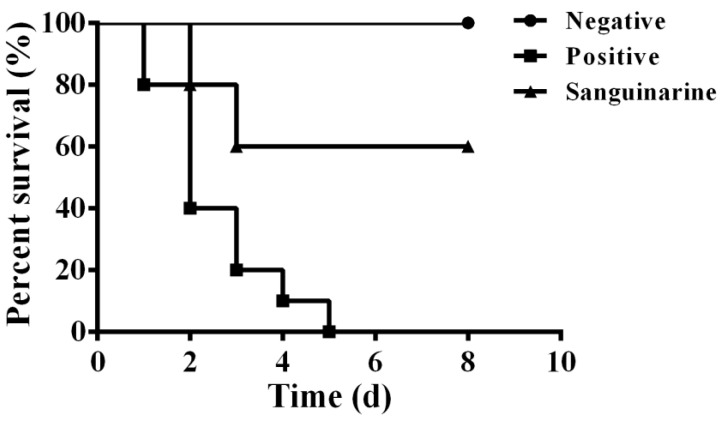
Sanguinarine protect channel catfish against *A. hydrophila* infection. Channel catfish (n = 10) were treated with 20 mg/kg sanguinarine post-infection, and survival of channel catfish were observed for 8 days.

In vitro studies demonstrated that sanguinarine could reduce the pathogenesis of *A. hydrophila* to A549 cells by inhibiting the secretion of aerolysin in sub-inhibitory concentrations, indicating that sanguinarine could reduce the mortality of channel catfish infected with *A. hydrophila*. Fish infected with *A. hydrophila* showed ulceration in body surface and swelling of the fins. Deaths were observed 24 h post-infection (Figure 6). *A. hydrophila* infection resulted in 100% mortality in 5 days, while this figure was 40% for the sanguinarine-treated group in 8 days. No deaths were observed in the negative group during the experimental period. The results demonstrate that sanguinarine could reduce the mortality of channel catfish challenged with *A. hydrophila*.

## 4. Discussion

The discovery of antibiotics has reduced the mortality of humans infected with bacterial pathogens [26]. Although a number of antibiotics have been identified, the emergence of antibiotic resistance by the abuse of antibiotics has brought great threats to human health [27]. *Aeromonas* spp. are susceptible to many antibacterial agents, such as quinolones, cephalosporin and chloramphenicol [28]. However, antibiotic resistance has been observed with the increasing use of antibiotics for the treatment of *A. hydrophila* infections in aquaculture [29]. Moreover, the resistance of *Aeromonas* spp. has been reported in strains isolated from clinical origin, food, and natural waters [28,30]. In the present study, the MIC value of enrofloxacin to *A. hydrophila* XS-91-4-1 was 4 μg/mL, indicating that the strain was resistant to enrofloxacin. Our previous experimental therapeutics assay demonstrated that the treatment of *A. hydrophila* XS-91-4-1 infection with enrofloxacin resulted in severe death in channel catfish [23]. The findings indicated that the emergence of antibiotic resistance limited the application of antibiotics in treating bacterial infections in aquaculture. Therefore, seeking novel strategies against resistant *A. hydrophila* infections is an urgent cause. With increasing knowledge of pathogenesis, the roles of virulence factors, adhesins and the quorum sensing system have been identified, and some of which have been identified as targets for developing drugs against *A. hydrophila* infections [31]. 

Pore-forming toxins are important virulence factors of pathogenic bacteria, which have often been identified as targets for developing drugs [32]. Aerolysin, the key virulence factor for pathogenic *A. hydrophila* strains, belongs to pore-forming toxins and plays a critical role in pathogenesis [33]. Therefore, aerolysin has been identified as an ideal target for developing drugs or vaccines against *A. hydrophila* infections [34]. In our present study, natural compounds isolated from traditional Chinese medicine were used to screen drugs against aerolysin production. 

Wang et al. found that sanguinarine had antibacterial activity against six bacterial pathogens, and *A.* *hydrophila* was the most susceptive among all the tested strains [35]. The MIC value of sanguinarine to *A. hydrophila* was 12.5 μg/mL according to the results by Kang et al. [36], while this was estimated to be around 32 μg/mL in the present study—higher than the value by Kang et al. [36]. The different sources of bacterial strains might result in a difference in MIC values. Ding et al. found that the MIC of sanguinarine against *Streptococcus agalactiae* was 8-16 μg/mL and the formation of bacterial biofilms could be inhibited [37]. Wang et al. investigated the inhibitory effects of sanguinarine against *Saprolegnia* sp. spores and mycelium, and the results showed that sanguinarine could provide an inhibition against both spores and mycelium, indicating that sanguinarine could be chosen as a potential fishery drug against *Saprolegnia* sp. infections [38]. Taken together, these findings indicated that sanguinarine had antimicrobial and anti-parasitic activities against fish pathogens in aquaculture. Moreover, several previous studies have demonstrated that sanguinarine could inhibit the growth of *Escherichia coli* (*E. coli*), *Staphylococcus aureus*, *Providencia rettgeri* and *Candida albicans* in vitro [39,40]. According to the results of the hemolytic assay, sanguinarine could significantly reduce the hemolytic activity of bacterial supernatant at a concentration of 1 μg/mL, and inhibit biofilm formation at a concentration of 0.5 μg/mL, while sanguinarine at this concentration had no role on bacterial growth. Taken together, sanguinarine could inhibit quorum sensing-mediated virulence factors without offering selective pressure to *A. hydrophila*. These findings indicate that treatment with sanguinarine could avoid promoting the emergence of resistant strains. 

The inhibitory effect of sanguinarine against bacterial pathogens has already been well studied. However, there was little knowledge available about the influence of sanguinarine on bacterial virulence. Zhang et al. reported that sanguinarine without anti-bacterial activity could decrease the production of the virulence proteins SipA and SipB in type III secretion systems, and could protect HeLa cells from *Salmonella enterica* serovar Typhimurium infection [41]. Tushar K Beuria demonstrated that sanguinarine could be identified as a leading compound in developing anti-bacterial agents by inhibiting FtsZ assembly and bundling [19]. These findings indicated that sanguinarine might be a candidate based on anti-virulence strategies. In the present study, we found that sanguinarine could significantly reduce the transcription of quorum sensing at concentrations higher than 1 μg/mL, and resulted in a decrease in pathogenesis both in vitro and in vivo. Ramanathan Srinivasan et al. found that naringin could be an anti-quorum sensing drug against *A. hydrophila* infections in *Danio rerio* [42]. However, the inhibitory concentrations of hemolysis and biofilm were higher than 375 μg/mL. The concentration could hardly be reached in vivo, which might decrease the therapeutic effect. Resveratrol was previously identified as an anti-virulence drug against *A. hydrophila* by inhibiting quorum sensing-mediated aerolysin production and biofilm; the inhibitory concentration was lower than sanguinarine, but a similar effect was achieved in experimental therapeutics [43]. Moreover, Bo Luo Hui San containing sanguinarine has been proven as a growth-promoting drug in aquaculture. Therefore, it is reasonable to believe that sanguinarine could be a promising candidate for treating bacterial infections in aquaculture. The inhibitory effect of sanguinarine not only provides a new approach to identifying drugs against *A. hydrophila* infections, but also partly explains the mode of action of herbal medicines. In the present study, sanguinarine was administered orally using a gavage needle, and achieved 60% survival rate. Because sanguinarine cannot fully inhibit the growth of *A. hydrophila* at 20 μg/mL, it is not recommended for immersion. Moreover, the experimental therapeutics only used 10 fish in each group, which might affect the reliability of the in vivo study. Thus, clinical trials using more testing fish are needed in future investigations. 

## 5. Conclusions

Sanguinarine could inhibit the production of aerolysin and the biofilm formation of *A. hydrophila* at sub-inhibitory concentrations. qPCR results indicated that sanguinarine could decrease the expression of aerolysin encoding gene *aerA*, and quorum sensing genes *ahyI* and *ahyR*. The results of the cell viability and experimental therapeutics demonstrated that sanguinarine could reduce the pathogenesis of *A. hydrophila* both in vitro and in vivo. The findings in the present study provide a plausible alternative for the treatment of *A. hydrophila* infections in aquaculture. 

## Figures and Tables

**Table 1:** Primer pairs used for real-time qPCR.

Primer	Sequence	PCR Amplicon (bp)
*aerA*-F	5′-TCTACCACCACCTCCCTGTC-3′	218
*aerA*-R	5′-GACGAAGGTGTGGTTCCAGT-3′	
*ahyI*-F	5′-GTCAGCTCCCACACGTCGTT-3′	202
*ahyI*-R	5′-GGGATGTGGAATCCCACCGT-3′	
*ahyR*-R	5′-CCTGGATGTCCAACTACATCTT-3′	206
*ahyR*-F	5′-TTTACGGGTGACCTGATTGAG-3′	
16S rRNA-F	5′-TAATACCGCATACGCCCTAC-3′	164
16S rRNA-R	5′-ACCGTGTCTCAGTTCCAGTG-3’	

## Data Availability

The data presented in this study are available on request from the corresponding author.
